# microRNA-152 Mediates DNMT1-Regulated DNA Methylation in the Estrogen Receptor α Gene

**DOI:** 10.1371/journal.pone.0030635

**Published:** 2012-01-25

**Authors:** Yung-Song Wang, Wen-Wen Chou, Ku-Chung Chen, Hsin-Yun Cheng, Ruey-Tay Lin, Suh-Hang Hank Juo

**Affiliations:** 1 Department of Medical Genetics, Kaohsiung Medical University, Kaohsiung, Taiwan; 2 Department of Neurology, Kaohsiung Medical University, Kaohsiung, Taiwan; 3 Department of Neurology, Kaohsiung Medical University Hospital, Kaohsiung, Taiwan; 4 Department of Medical Research, Kaohsiung Medical University Hospital, Kaohsiung, Taiwan; 5 Cancer Center, Kaohsiung Medical University Hospital, Kaohsiung, Taiwan; Bellvitge Biomedical Research Institute (IDIBELL), Spain

## Abstract

**Background:**

Estrogen receptor α (ERα) has been shown to protect against atherosclerosis. Methylation of the ERα gene can reduce ERα expression leading to a higher risk for cardiovascular disease. Recently, microRNAs have been found to regulate DNA methyltransferases (DNMTs) and thus control methylation status in several genes. We first searched for microRNAs involved in DNMT-associated DNA methylation in the ERα gene. We also tested whether statin and a traditional Chinese medicine (San-Huang-Xie-Xin-Tang, SHXXT) could exert a therapeutic effect on microRNA, DNMT and ERα methylation.

**Methodology/Principal Findings:**

The ERα expression was decreased and ERα methylation was increased in LPS-treated human aortic smooth muscle cells (HASMCs) and the aorta from rats under a high-fat diet. microRNA-152 was found to be down regulated in the LPS-treated HASMCs. We validated that microRNA-152 can knock down DNMT1 in HASMCs leading to hypermethylation of the ERα gene. Statin had no effect on microRNA-152, DNMT1 or ERα expression. On the contrary, SHXXT could restore microRNA-152, decrease DNMT1 and increase ERα expression in both cellular and animal studies.

**Conclusions/Significance:**

The present study showed that microRNA-152 decreases under the pro-atherosclerotic conditions. The reduced microRNA-152 can lose an inhibitory effect on DNA methyltransferase, which leads to hypermethylation of the ERα gene and a decrease of ERα level. Although statin can not reverse these cascade proatherosclerotic changes, the SHXXT shows a promising effect to inhibit this unwanted signaling pathway.

## Introduction

Atherosclerosis is the major pathogenesis of cardiovascular disease that causes morbidity and mortality in industrialized countries [1]. This progressive multi-factorial process involves cellular signaling pathways [2]–[4], inflammatory mediators, growth factors, and adhesion molecules [5], [6]. Risk factors such as unhealthy dietary habits, aging and smoking contribute to the development of cardiovascular disease through increasing blood lipid levels and inflammation in the arterial wall. Eventually, arterial smooth muscle cells migrate and proliferate leading to atherosclerotic plaque formation and atherosclerosis development [7], [8]. Recently, DNA methylation has been implicated as a novel risk factor for atherosclerosis [9], [10].

DNA methylation is an important epigenetic modification on chromosomes that plays a significant role in the regulation of gene transcription [11]. When the cytosine and guanine contents are greater than 50% in the DNA sequence of humans, the high CG content regions can be hypermethylated causing transcriptional silencing [12]. Increasing evidence shows that global DNA hypomethylation is associated with gene transcriptional activity in the pathogenesis of cardiovascular diseases, including atherosclerosis [13]–[16]. Hiltunen et al. reported global DNA hypomethylation in the proliferating vascular smooth muscle cells (VSMCs) of human atherosclerotic plaque [9].

Estrogen receptor α (ERα) has been reported to protect against atherosclerosis and aging in the cardiovascular system [17], [18]. Increased expression of the ERα gene can reduce the proliferation of VSMCs [19], [20]. DNA methylation in the promoter region of the ERα gene can reduce transcription of ERα leading to a higher risk for several cardiovascular diseases [18].

It is known that DNA methylation is catalyzed by DNA methyltransferases (DNMTs), namely DNMT1, DNMT3a and DNMT3b. Methytransferases use a reactive methyl group bound to sulfur in S-adenosyl methionine as the methyl donor. DNMT1 is the most abundant DNA methyltransferase in mammalian cells and the key maintenance enzyme for hemimethylated DNA during DNA replication of various cancer cells [21]. DNMT3a and DNMT3b are responsible for establishing *de novo* methylation and both enzymes exist in diverse cancer cells and cell lines [12], [22]. Both recent evidence and our previous study showed that microRNAs (miRs), which are noncoding RNAs, can be involved in the promoter methylation of CpG islands by targeting DNMTs 3′UTR [23]–[27]. Thus, we first aimed to search for miRNAs involved in DNMT-associated DNA methylation in the ERα gene.

Statin is a widely used lipid-lowering drug with multiple effects on the cardiovascular system [28]. Statin has been shown to have multiple mechanisms to reduce the cardiovascular risk including the anti-inflammation effects [29] and the inhibition of neointimal proliferation [30], [31]. We previously also found San-Huang-Xie-Xin-Tang (SHXXT), a traditional Chinese medicine, has several beneficial anti-atherosclerotic and anti-inflammatory effects [32]. In the present study, we also tested whether statin and SHXXT can regulate DNA methylation in the ERα gene as our second aim.

## Results

### ERα expression in LPS-treated HASMCs and HF feeding in rats

**Figure 1:**
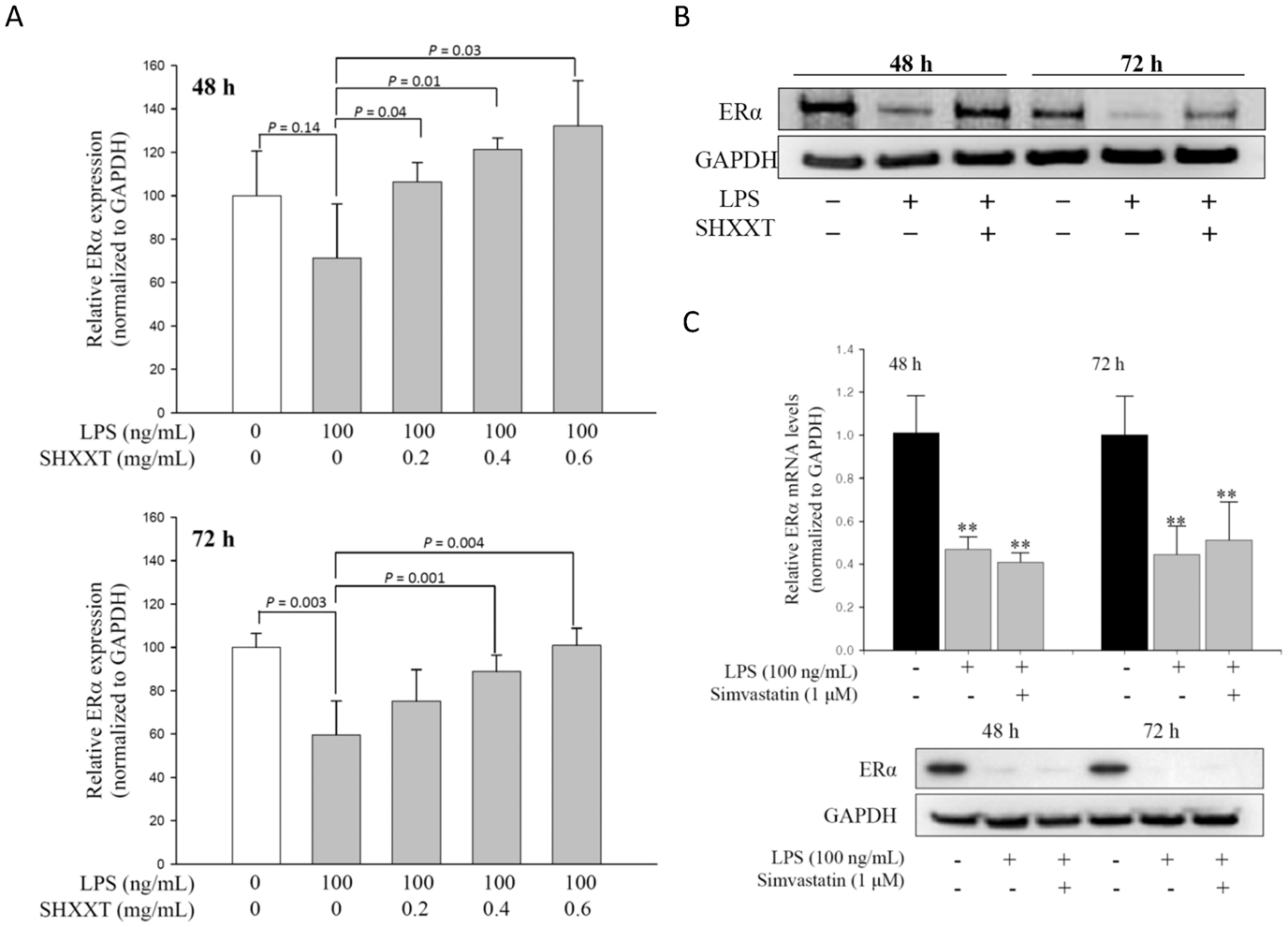
mRNA and protein expression of ERα in the HASMCs treated with LPS, and the change of promoter methylation in HASMCs. (A) HASMCs were treated with LPS (100 ng/mL) and SHXXT (0.2–0.6 mg/ml) for 48 h and 72 h. The mRNA expression of ERα after LPS and SHXXT treatment was detected by real time PCR and normalized to the housekeeping gene GAPDH, and (B) the protein level of ERα after LPS and SHXXT treatment was detected by Western blot and normalized to the housekeeping gene GAPDH. (C) ERα mRNA and protein level after simvastatin (1 µM) treatment were detected by real-time PCR and Western blot at 48 h and 72 h. ** *P*<0.01 versus the control group. Data represents mean ± SD from 3 independent experiments performed in triplicates.

**Figure 2:**
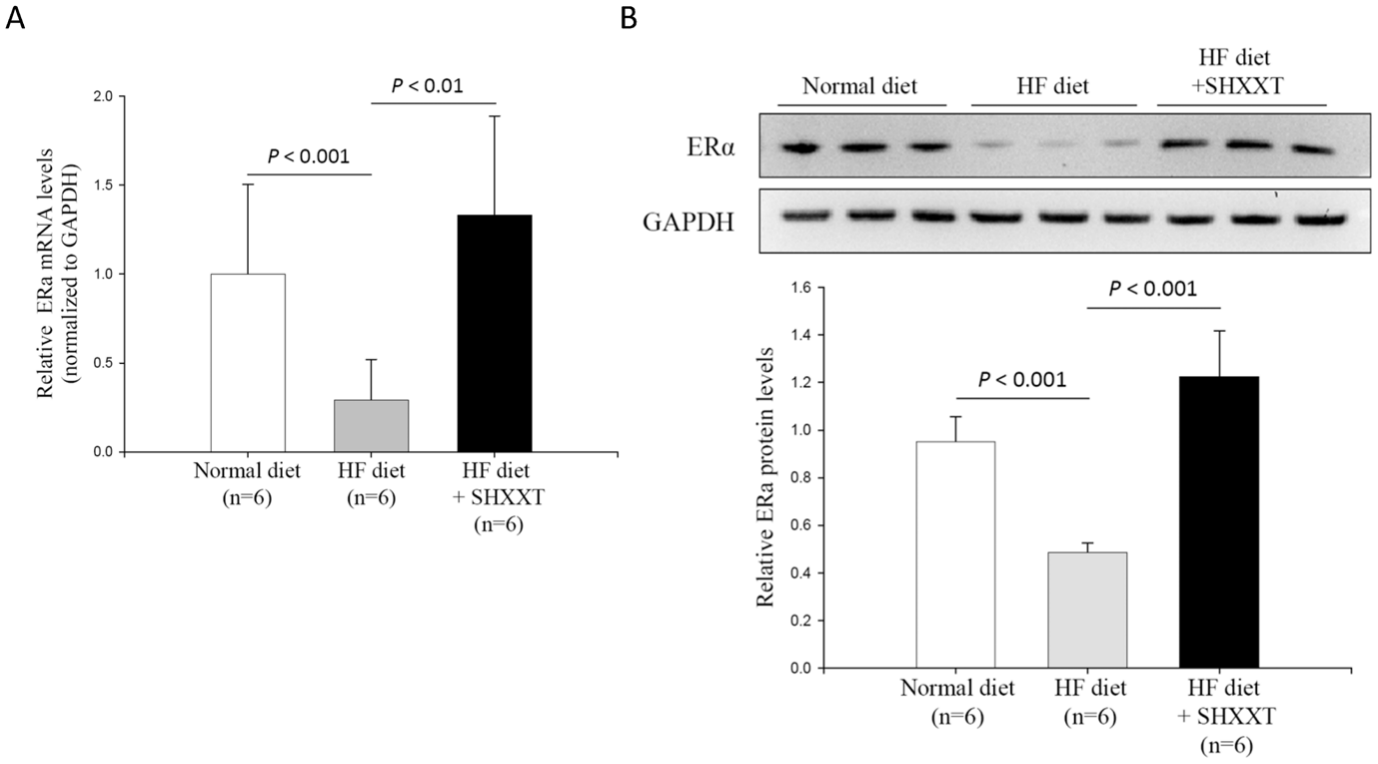
The expression of ERα in the aorta of the Sprague-Dawley rats under a high fat diet. In addition to a normal diet, rats under a high fat diet were treated with SHXXT (30 mg/kg/day) or placebo (water) for 12 weeks. (A) The ERα mRNA expression of each group (n = 6) was detected by real time PCR and normalized to GAPDH. Data represents mean ± SD. (B) The protein level of ERα of each group was detected and normalized to GAPDH. The number of SD rat in each group is 6. Data represents mean ± SD.

In our previous study, LPS (100 mg/ml) significantly induced the cell proliferation, migration and inflammation. In this study, we first found that LPS treatment significantly decreased ERα mRNA level and protein level at 48 h and 72 h (Fig. 1A and 1B). Furthermore, ERα expression can also be restored by SHXXT in a dose-dependent manner (Fig. 1A and 1B). However, simvastatin (1 µM) treatment had no effect on the decreased ERα at 48 h or 72 h (Fig. 1C). In order to confirm that the SHXXT effect on increasing ERα expression, we compared the mRNA and protein expression of the ERα gene in the aorta between the HF diet-fed SD rats with and without treatment of SHXXT. After 12 weeks, the ERα mRNA and protein levels were decreased in the HF diet-fed group as compared with the normal diet group (Fig. 2A and 2B).

### DNMT1 expression in LPS-treated HASMCs

For the global methylation pattern, we found that the proportion of methylation in the Alu elements maintained at ∼16% in HASMCs (measured at 48 h and 72 h) prior to LPS treatment. The proportion of methylation was substantially decreased to 5% at 72 h, although it was only decreased from 16% to 13% at 48 h after LPS treatment.

**Figure 3:**
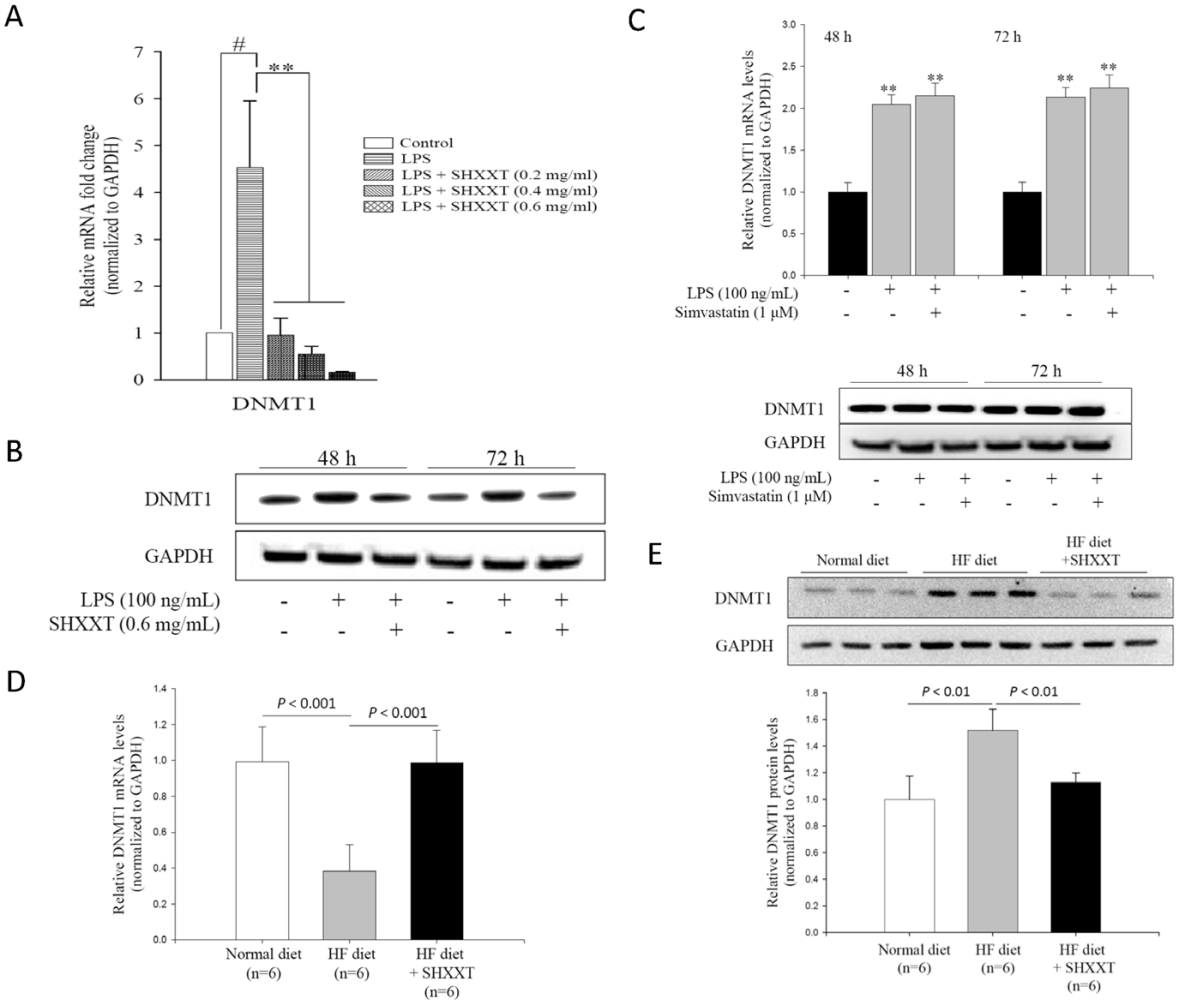
DNMT1 expression in LPS-induced HASMCs and the aorta of the rats under a high fat diet. (A) HASMCs were treated with LPS (100 ng/mL) and SHXXT (0.2–0.6 mg/ml) for 48 h. DNMT1 mRNA expression were examined by real-time quantitative PCR and normalized to RU6B. (B) DNMT1 protein in LPS-treated HASMCs measured by Western blot at 48 h and 72 h. (C) DNMT1 mRNA and protein level after the treatment of simvastatin for 48 h and 72 h. ** *P*<0.01 versus the control group. Data represents mean ± SD from 3 independent experiments performed in triplicates. (D and E) The mRNA expression and protein level of DNMT1 of the rat aorta under a normal diet and a high-fat diet with or without SHXXT (30 mg/kg/day) for 12 weeks. The upper panel of Figure 3E shows the western blotting data and lower panel shows the quantification of western blotting. The number of SD rat in each group is 6. ^#^ *P*<0.05 versus control, ** *P*<0.01 versus LPS group. Data represents mean ± SD.

Since DNMT1 is the major enzyme for DNA methylation in mammals, we focused on this DNMT and tried to elucidate the regulation mechanism. As shown in Fig. 3A and 3B, the increases of DNMT1 mRNA expression and protein levels in HASMCs were found at 48 h and 72 h after the treatment of LPS. When adding SHXXT (dose between 0.2 to 0.6 mg/ml), the mRNA and protein expression of DNMT1 were substantially reduced after incubation for 48 h and 72 h. On the contrary, neither mRNA nor protein level changed by treating simvastatin (Fig. 3C), which suggests simvastatin does not influence DNMT1-associated DNA methylation. Similar to the results from the cellular studies, DNMT1 expression in the aorta of SD rats was significantly increased in the HF-diet group and decreased by SHXXT (Fig. 3D and 3E). Therefore, we demonstrated a consistent change between ERα and DNMT1, and found a potential therapeutic effect of SHXXT.

### Gain of ERα by DNMT1 knockdown

**Figure 4:**
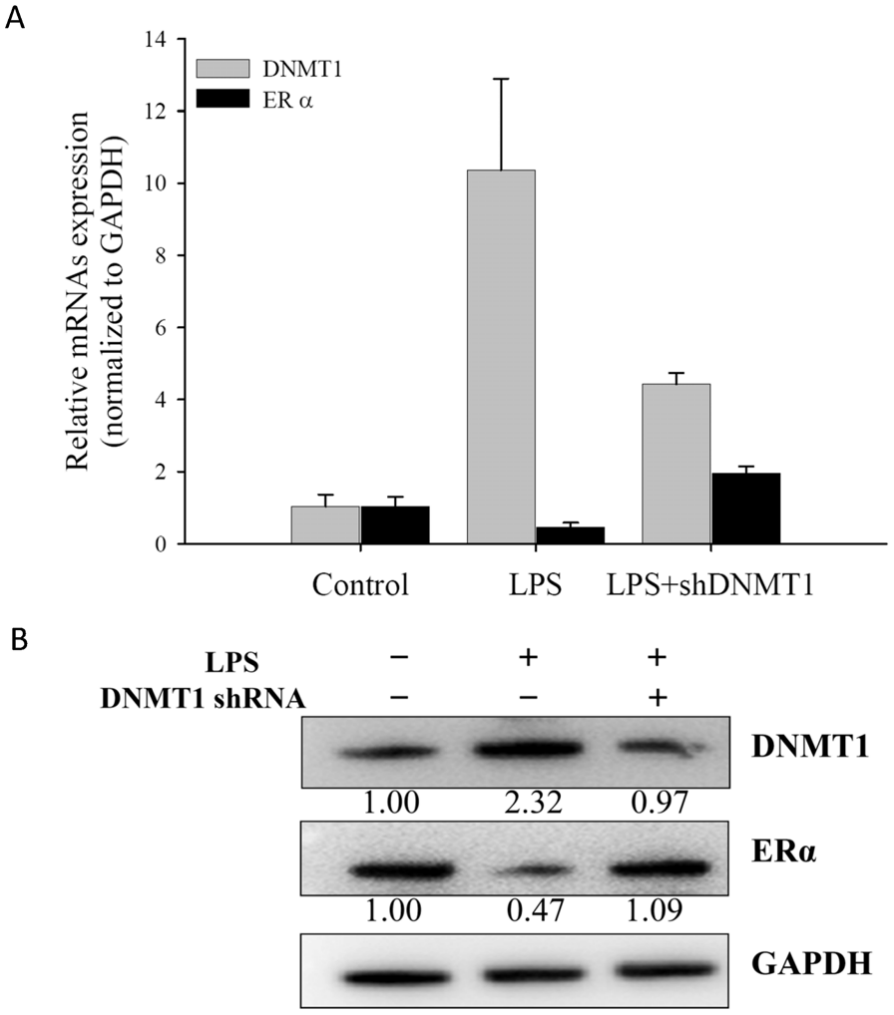
Expression of ERα after knocking down DNMT1. LPS-treated HASMCs were transfected with shRNAs (6 µg DNA per 10^5^ cells) specifically targeting DNMT1. The data were obtained at 48 h post-transfection of shRNAs. (**A**) The mRNA levels of DNMT1 and ERα were examined with real-time quantitative PCR. Data represents mean ± SD. (**B**) Protein levels of DNMT1 and ERα were examined by specific antibody.

To further confirm that DNMT1 can influence ERα expression, we used shRNA to knock down the DNMT1 gene. The RNA inference caused a decrease of DNMT1 mRNA expression and protein expression by 57.3% and 58.2% respectively (Fig. 4A and 4B). When DNMT1 was inhibited, the mRNA and protein levels of ERα were simultaneously increased by 4.33- and 2.32-fold, respectively (Fig. 4A and 4B). These results clearly show that ERα gene can be silenced by DNMT1.

### ERα methylation in LPS-treated HASMCs and HF fed rats

**Figure 5:**
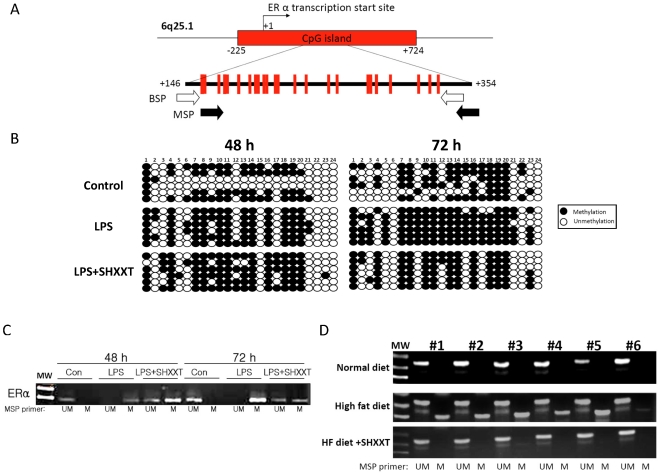
Methylation of the ERα gene in HASMCs induced by LPS with or without SHXXT treatment. (A) Schematic diagram of the distribution of ERα exon I CpG islands in HASMCs (GenBank accession no. X03635.1 G1:31233) are shown. Black and white arrows indicated the PCR primer pair of methylation specific PCR (MSP) and bisulfite sequencing PCR (BSP), respectively. (B) Exon 1 of the ERα methylation status was analyzed by MSP at 48 h and 72 h. Electrophoresis showed methylation-specific PCR products from three conditions (Control, LPS treatment and LPS plus SHXXT treatment).UM: unmethylated; M: methylated ERα. (C) PCR primers specific to unmethylated and methylated bisulfite-modified DNA were used to amplify exon 1 of the ERα gene in LPS-induced HASMCs and SHXXT treatment. Amplified PCR products were cloned, and six individual clones were sequenced to determine the methylation state of the 24 CpGs within the amplified region. Open and filled circles indicate that the CpG site is unmethylated or methylated, respectively. Each row represents one clone. (D) Exon 1 of the ERα methylation status of the rat aortas were analyzed by MSP. UM: unmethylated; M: methylated ERα.

To determine if ERα locus undergoes promoter hypermethylation in LPS-treated HASMCs, we checked the methylation status of the CpG island located in the first exon of ERα gene (Fig. 5A) by the bisulfite specific PCR and sequencing (BSP) as well as methylation-specific PCR (MSP). We first measured the methylation degree of 24 CpG sites in exon 1 (nucleotides 146–354) by the BSP assay [33]. At 48 h and 72 h after LPS treatment, the methylation percentage was increased (57% and 77%, respectively) as compared with the control group (40% and 49%, respectively; Fig. 5B) in overall 24 CpG sites. Although we also found that the DNA methylation status of ERα can be reversed by SHXXT treatment, but the drug effect mainly occurred at 72 h (55%). In the MSP assay, we found that DNA methylation status in the ERα exon 1 was also increased by LPS, and SHXXT partially reversed LPS-induced DNA hypermethylation at 48 h and 72 h (Fig. 5C). These data suggested that ERα exon 1 hypermethylation does occur in LPS-induced HASMCs and affect ERα expression. In order to confirm that the SHXXT effect on reducing ERα hypermethylation, we compared the methylation status of the ERα gene in of the aorta between the HF diet-fed SD rats with and without treatment of SHXXT. After 12 weeks, SHXXT could significantly reverse HF diet-induced DNA hypermethylation of the ERα gene (Fig. 5D).

### miR-152 expression in LPS-treated HASMCs

**Figure 6:**
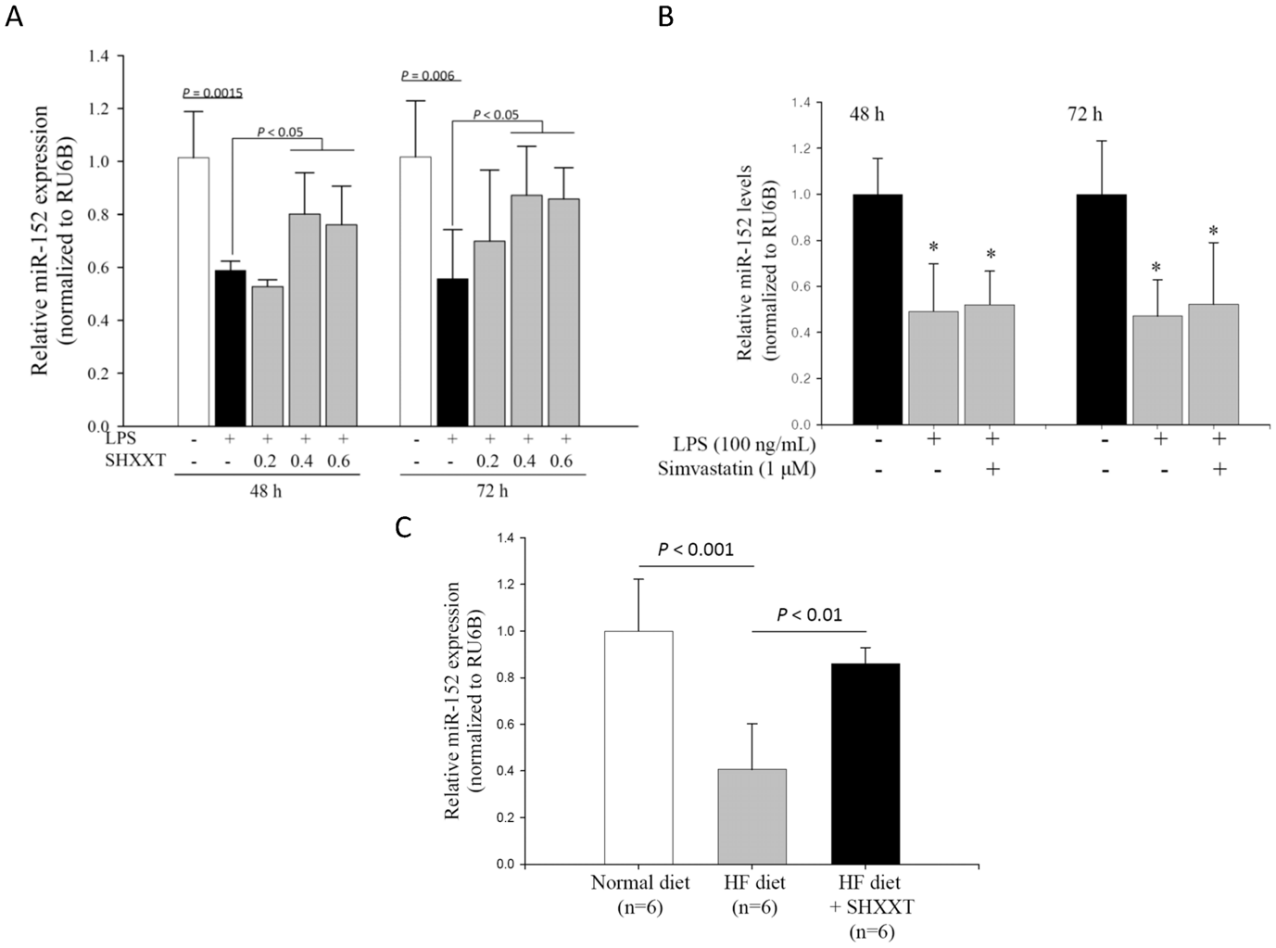
Expression of miR-152 in LPS-treated HASMCs and the aorta of the Sprague-Dawley rats. (A) The miR-152 levels in the LPS -treated HASMCs were examined by real-time quantitative PCR and normalized to RU6B after 48 h and 72 h treatment with/without SHXXT. (B) miR-152 expression were detected by real-time quantitative PCR after treatment with simvastatin (1 µM). * *P*<0.05 versus the control group. Data represents mean ± SD from 3 independent experiments performed in triplicates. (C) miR-152 expression of the rat aorta evaluated by real-time quantitative PCR. Rats under a high fat diet were treated with SHXXT (30 mg/kg/day) or placebo (water) for 12 weeks. The SD rat number of each group is 6. Data represents mean ± SD.

To identify the atherosclerosis-related miRNAs, we used the miRNA arrays (OneArray microRNA expression profiling microarrays based on the latest miRBase release-version 17; Phalanx Biotech Group, Taiwan) to survey the miRNA expression levels between HASMCs treated and untreated with LPS (100 ng/mL). This commercial array used spotted probes to interrogate 1087 human miRNAs. Among 1,087 surveyed miRNAs, the expression level of miR-152 was decreased by 2.3-fold when HASMCs were treated with LPS for 24 h (data not shown). Subsequent real-time PCR experiment confirmed that LPS can decrease miR-152 expression at 48 h and 72 h (*P* = 0.0015 and 0.006; Fig. 6A). Previous studies on cancers [23], [24] reported that miR-152 can bind to the 3′ untranslated region (UTR) of DNMT1. Accordingly, we speculated that miR-152 may be involved in DNMT1-associated DNA methylation in the cardiovascular system. Furthermore, our cellular studies showed that miR-152 levels can be induced by SHXXT in a dose-dependent manner, but cannot be induced by simvastatin for 48 h or 72 h (Fig. 6B). In the aorta of the SD rats, we confirmed that the miR-152 expression was down-regulated in the HF-diet group, and it can be up-regulated after 12-week treatment of SHXXT (n = 6; Fig. 6C).

### Gain and loss functions of ERα by miR-152

**Figure 7:**
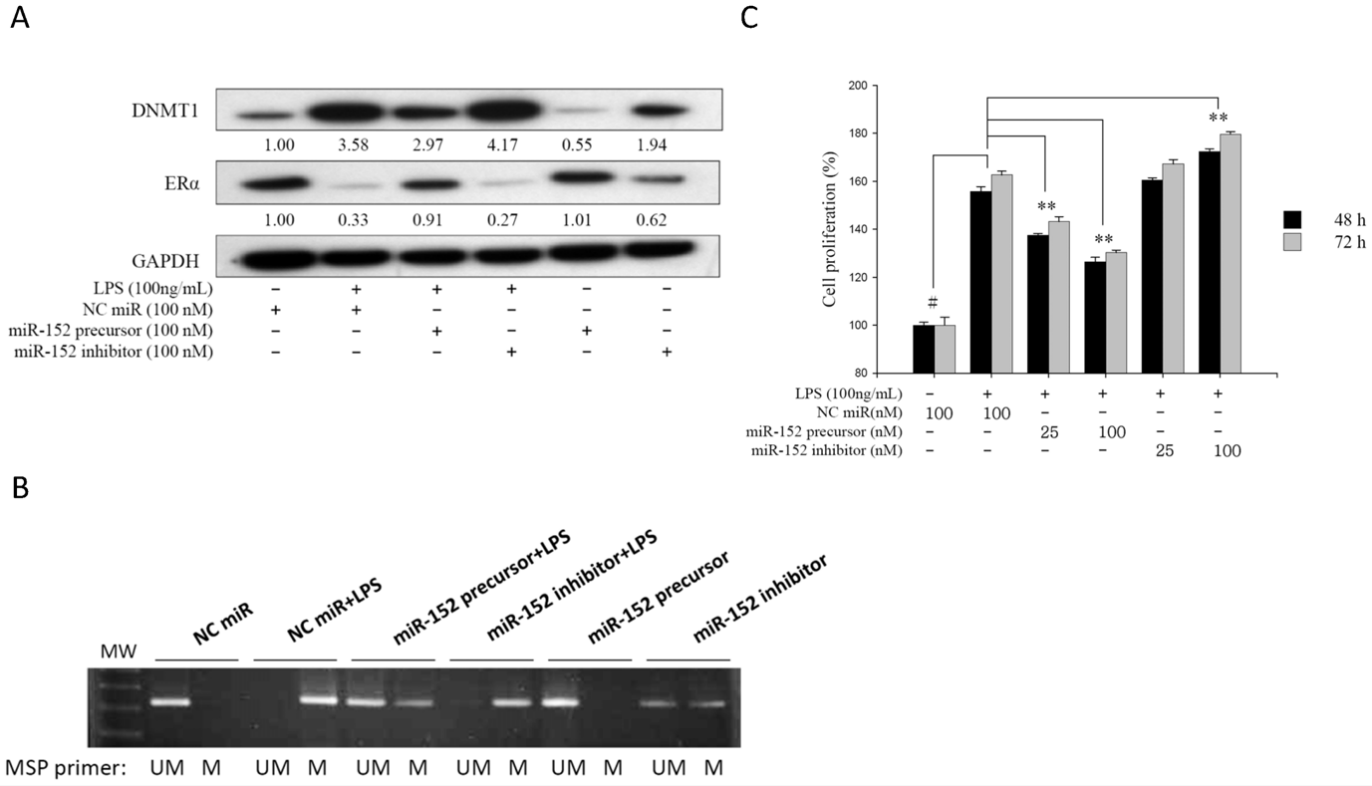
miR-152 regulates DNMT1 and ERα at the protein levels. LPS-treated HASMCs were transfected with miR-152 precursor/inhibitor (25 nM and 100 nM) and a negative control miR (NC miR, 100 nM). (A) DNMT1 and ERαprotein levels were measured by Western blot at 72 h post-transfection. (B) Exon 1 of the ERα methylation status was analyzed by MSP at 72 h post-transfection. UM: unmethylated; M: methylated ERα. (C) HASMCs were incubated with LPS with the miR-152 precursor or inhibitor for 48 h and 72 h and the cell proliferation was assessed using the MTT assay to measure mitochondrial enzyme activity. ^#^ *P*<0.01 versus control, **P<0.01 versus the LPS group. Data represents mean ± SD from 3 independent experiments performed in triplicates.

To prove that miR-152 can regulate ERα gene expression via the suppression of DNMT1, we transfected the miR-152 precursor or inhibitor into the LPS-treated HASMCs. The results showed that the DNMT1 protein levels were reduced and consequently ERα protein levels were up-regulated by miR-152 precursor at 72 h (100 nM; Fig. 7A). On the contrary, DNMT1 protein levels were increased and consequently ERα protein levels were down-regulated by miR-152 inhibitor at 72 h (100 nM). These findings indicate that miR-152 indirectly up-regulated ERα expression through its binding to DNMT1. MSP analysis also showed that DNA methylation at ERα was reduced by miR-152 precursor in the LPS-treated cells (Fig. 7B). However, DNA methylation at ERα was increased by miR-152 inhibitor. Therefore, we demonstrated a consistent change between DNMT1 and ERα methylation by the change of miR-152 levels.

### The effect of miR-152 on HASMC proliferation and migration

To further examine whether miR-152 influences HASMC phenotypes through the change of ERα expression, the cell proliferation and migration were assessed at 48 h and 72 h after transfecting miR-152 precursor or inhibitor into the cells. The results demonstrated that the transfected miR-152 precursor resulted in a concentration-dependent reduction of cell proliferation in the LPS-treated HASMCs at 48 h and 72 h (Fig. 7C; *P*<0.01). However, miR-152 did not influence HASMC migration at 48 or 72 h (data not shown). These results also suggested that miR-152 has an anti-atherosclerotic effect by decreasing cell proliferation.

## Discussion

Increasing evidence has revealed that the ERα protein has an atheroprotective effect and methylation in the ERα gene can cause atherosclerosis [17], [18]. However, the role of microRNAs in the regulation of ERα expression has not been extensively explored. The present study showed that miR-152 can down regulate DNMT1 which in turn inhibits methylation in the promoter of the ERα gene leading to higher ERα expression. Therefore, we identified that miR-152 has an anti-atherosclerotic effect via its effect on the increase of ERα expression. We also used LPS to induce hypermethylation of the ERα gene in HASMCs. Although statin failed to reverse the hypermethylation status in the ERα gene, the traditional Chinese medicine (SHXXT) partially recovered LPS-induced DNA hypermethylation. Furthermore, our animal studies confirmed the therapeutic effect of this Chinese medicine. Again, statin did not have any effect on increasing miR-152 but SHXXT could enhance miR-152 expression. Unfortunately, we did have available human samples to compare miR-152 concentrations between atherosclerotic and non-atherosclerotic tissues. Taken together, the present study not only identified a novel mechanism to regulate ERα expression but also provided data for the Chinese medicine SHXXT in the context of epigenetic therapy in the context of atherosclerosis and cardiovascular disease.

It is well known that estrogen has directly protective effect on the cardiovascular system [17], [34], [35]. Two types of ER (α and β subtypes) have been identified in VSMCs and endothelial cells. Several studies have shown that estrogen inhibits the proliferation and migration of VSMCs via ERα binding [17], [35], [36]. Furthermore, the anti-atherosclerotic effects of ERα also can be influenced by the methylation status in its promoter region [18], [19]. Our data provided more insight to how the ERα gene is regulated by epigenomic mechanism and microRNA.

Newman [37] first proposed that DNA hypomethylation is involved in the development of cardiovascular disease. Increased plasma homocysteine and S-adenosylhomocysteine (SAH) are associated with the inhibition of methyltransferases, which causes global DNA hypomethylation in VSMCs [38], [39], peripheral white blood cells [39] and atherosclerotic lesions [40]. More solid evidence from animal studies suggests that cellular proliferation is associated with global DNA hypomethylation and DNMT1 overexpression in atherosclerotic lesions [39]. Theoretically, over-expressed DNMT1 should cause DNA hypermethylation, but the paradoxical finding of increased global DNA hypomethylation and DNMT1 overexpression was reported in previous studies [39]. Therefore, there must be other mechanisms regulating DNA methylation, and in the present study we cannot rule out the involvement of these undiscovered mechanisms in ERα hypermethylation. Indeed, co-existence of global hypomethylation and hypermethylation in particular genes have been reported in the atherosclerosis [13]. The DNMT family can be regulated by several miRs such as miR-29b, miR-148a and miR-152 [23]–[25], [27]. Given that epigenetic therapy has been advertised, our finding of SHXXT's effect on influencing microRNA and DNA methylation may offer a different approach in conjunction with statin for a better treatment for cardiovascular diseases.

In conclusion, the present study showed that the level of miR-152 decreases under the pro-atherosclerotic stimulations. The reduced miR-152 can lose an inhibitory effect on DNA methyltransferase, which contributes to hypermethylation of the ERα gene. Once hypermethylation takes place in the promoter of the ERα gene, atherosclerosis can be promoted. Although statin cannot reverse these cascade proatherosclerotic changes, the Chinese medicine, SHXXT, shows a potential effect to inhibit such an unwanted signaling pathway.

## Materials and Methods

### HASMC culture and treatment

Human aortic smooth muscle cells (HASMCs, cryopreserved tertiary cultures; Cascade Biologics, Inc.,OR, USA) were grown in culture flasks in smooth muscle cell growth media (Medium 231, Cascade Biologics, Inc., OR, USA). The smooth muscle cell growth medium consisted of fetal bovine serum (FBS, 5%), human epidermal growth factor (10 ng/ml), human basic fibroblast growth factor (3 ng/ml), insulin (10 mg/ml), penicillin (100 units/ml), streptomycin (100 pg/ml), and Fungizone (1.25 mg/ml). The cells were incubated at 37°C in a humidified 5% CO2 atmosphere. HASMCs were subcultured when the cells were about 70–80% confluence. Passages 4 to 9 were used for experiments. Lipopolysaccharide (LPS; 100 ng/mL) was used to induce an atherogenic change in HASMCs. Before LPS treatment, cells were grown under HASMC medium without FBS for 24 h. Cells were then incubated at 37°C for 24 h in the presence of LPS. SHXXT contains three components: *Coptidis rhizome* (rhizomes of *Coptis chinesis* Franch; CR), *Scutellariae radix* (roots of *Scutellaria baicalensis* Georgi; SR), and *Rhei rhizome* (rhizomes of *Rheum officinale* Baill; RR). SHXXT was prepared by as follows: powdered crude extracts of RR, SR and CR were weighed in a ratio of 2: 1: 1. Each extract was added to 10-fold volume of water and then boiled in different conditions individually. After finishing the extraction, the materials were filtered to yield three extraction solutions. Three hot solutions were combined and then condensed to 1/20 in volume of the total original water. The new formed condensed solution was spray-dried to get the final dry SHXXT. SHXXT was dissolved in deionized distilled water along with LPS for a 24 h incubation period. Simvastatin (1 µM; Sigma-Aldrich, MO, USA) was dissolved in ETOH and was subsequently diluted in saline. Both SHXXT and simvastatin were tested for the effect on DNA methylation of the ERα gene in the HASMCs.

### Animal Preparation

Eight-week-old Sprague-Dawley male rats were divided to three groups (normal diet, high fat (HF) and HF plus SHXXT). SHXXT (30 mg/kg/day) or placebo (water) was orally fed using the ball-tipped feeding needle for 12 weeks. Rats were raised in a controlled environment at 20±2°C, with 40–70% relative humidity and an artificial 12-h light-dark cycle. All animal experiments were carried out in compliance with the “Guide for the Care and Use of Laboratory Animals” published by the National Academy of Sciences. The protocol was approved by the Kaohsiung Medical University Animal Research Committee (IACUC approval No.98018). After acclimatization for 1 week, eighteen rats were randomly divided into 3 groups. The composition of the HF diet was enriched with 30% fat. Access to food and water was unrestricted for either group. The body weight, food and water consumption of the animals were measured daily. Rats were killed at the end of 12th week. Their aortas were removed and immediately frozen in liquid nitrogen until assayed.

### PCR of Alu repetitive elements

Methylation analysis of Alu repetitive elements was performed by the COBRA assay as described previously [41]. Methylation quantification was performed with a molecular Dynamics PhosphorImager.

### Methylation specific PCR and sequencing

DNA was extracted from HASMCs and rat aortas by the DNA extraction kit (Qiagen, CA, USA). Next, bisulfite conversion was performed using the EZ DNA methylation Gold kit (Zymo research, CA, USA) according to the manufacturer's instructions. Methylation specific PCR was then carried out to determine the methylation status of ERα. Bisulphite-modified DNA was used for PCR with primers specifically designed for methylated or unmethylated sequences (Table 1). The PCR conditions were as follows: 95°C for 5 min followed by 40 cycles of 95°C for 1 min, 56°C for 1 min and 72°C for 2 min, and then a final extension at 72°C for 7 min. PCR products were analyzed by electrophoresis in a 2.0% agarose gel containing ethidium bromide.

**Table 1:** Primer sets.

Species	Gene	Primer Sequence
Human	MSP for ERα	Unmethylated-specific pair
		F: 5′-TGTTGTGTATAATTATTTTGAGGGT-3′
		R: 5′-CTCACACACCATATAACCACTAAAC-3′
		Methylated-specific pair
		F: 5′-CGTCGTGTATAATTATTTCGAGGGC-3′
		R: 5′-CTCGCGCACCGTATAACCGCTAAAC-3′
	BSP for ERα	First round pair
		F: 5′-ATGGTTTTATTGTATTAGATTTAAGGGAAT-3′
		R: 5′-TATTACICTAAACTCITTCTCCAAATAATA-3′
		Second round pair
		F: 5′-AGTGTATTTGGATAGTAGTAAG-3′
		R: 5′-CTAACCITAAAACTACAAAAAAAA-3′
	PCR for	
	DNMT1	F: 5′-GCACAAACTGACCTGCTTCA-3′
		R: 5′-GCCTTTTCACCTCCATCAAA-3′
	ERα	F: 5′-TTCGGCTCCAACGGCCTGGGGGGTTT-3′
		R: 5′-GGTACTGGCCAATCTTTCTCTGCCACCCT-3′
	GAPDH	F: 5′-GAAGGTGAAGGTCGGAGTC-3′
		R: 5′-GAAGATGGTGATGGGATTTC-3′
SD rat	MSP for ERα	Unmethylated-specific pair
		F: 5′-TGTTGTTGTTGTGTGGTTGTTGG-3′
		R: 5′-TCAACAAACTAAACAACACAC-3′
		Methylated-specific pair
		F: 5′-TTAATTATTTCGAGGGCGTC-3′
		R: 5′-AAAACGACGACACGTACGAC-3′
	PCR for	
	DNMT1	F: 5′-ACCTACCACGCCGACAT-3′
		R: 5′-AGGTCCTCTCCGTACTCCA-3′
	ERα	F: 5′-GGCTGCGCAAGTGTTACGAA-3′
		R: 5′-CATTTCGGCCTTCCAAGTCAT-3′
	GAPDH	F: 5′-CCTTCATTGACCTCAACTAC-3′
		R: 5′-GGAAGGCCATGCCAGTGAGC-3′

Bisulfite sequencing of HASMC DNA samples was performed as described previously [33]. The bisulfite-modified DNA was used to amplify a 208-bp product of ERα exon 1 gene with primer sets mentioned at Table 1. Two stage PCR were used to amplify a region with in the ERα CpG island (GenBank accession no. X03635.1 G1:31233). PCR products were cloned using pGEM-T Vector System according the manufacturer's instructions (Promega). The sequenced DNA region for ERα was confirmed by using the automated sequencer (ABI automated DNA sequencer).

### RNA interference

We used shRNA to perform RNA interference. The lenti-viral plasmids expressing 21-mer shRNAs against DNMT1 (clone ID: TRCN0000021890) was obtained from the National RNAi Core Facility in the Academia Sinica (Taipei, Taiwan). HASMCs were transfected with the empty vector (pLKO.1 puro) or plasmids expressing shRNA (6 µg DNA per 10^5^ cells for 48 h) using Lipofectamine 2000 (Invitrogen, CA, USA).

### MicroRNA transfection

A negative control miRNA (#17110), hsa-miR-152 precursor (Product ID: PM12269) and hsa-miR-152 inhibitor (Product ID: AM12269) were purchased from the Ambion Inc. (TX, USA). HASMCs were transfected with miRNA precursor using Lipofectamine 2000.

### RNA isolation, cDNA synthesis, and quantitative real-time PCR

Total RNA was isolated from HASMCs and rat aortas using Trizol reagent (Invitrogen, CA, USA). cDNA was synthesized by reverse transcription with 1 µg RNA using the SuperScript™ kit (Invitrogen, CA, USA). The 20 µl reverse transcription products were diluted to 100 µl and 2 to 3 µl was used for real-time PCR. Real-time PCR reactions were performed in a volume of 25 µl containing 140 ng specific primers (Table 1) and 12.5 µl SYBR green Master Mix (ABI, CA, USA) using the following condition: Initial denaturing: 95°C for 10 min, followed by 40 cycles of denaturing at 95°C for 30 s, annealing at 56°C for 40 s, and extension at 72°C for 30 s. The threshold cycle number (CT) value for the target gene (DNMT1 and ERα) was normalized against GAPDH and calculated as ΔCT = CT^Target^−CT^GAPDH^. Expression levels of mRNA were quantified by employing the 2^−ΔΔCt^ relative quantification method. Real-time PCR was performed for Hsa-miR-152 (UCAGUGCAUGACAGAACUUGGG; Assay ID: 000438) using the specific TaqMan MiRNA Reverse Transcription kit (Applied Biosystems). All the reactions were amplified on a 7900 HT Fast Real Time PCR system (Applied Biosystems). Expression levels of miRNA were quantified employing the 2^−ΔΔCt^ relative quantification method. The threshold cycle number (CT) value for miR-152 was normalized against RU6B and calculated as ΔCT = CT^miR-152^−CT^RU6B^. Expression levels of miRNA were quantified by employing the 2^−ΔΔCt^ relative quantification method.

### Western blot for DNMT1 and ERα

HASMCs were homogenized in 100 µl of protein extraction reagent (Thermo Scientific, MA, USA) and protease inhibitor (Panomics, CA, USA). Protein concentration was determined by Pierce BCA Protein Assay Kit (Thermo Scientific, MA, USA). 20 µg of protein was loaded per lane and separated by NuPAGE Novex Bis-Tris 4–12% mini gel electrophoresis (Invitrogen, CA, USA) in the Novex Xcell-II apparatus for 120 min at 100 V, and transferred to Immbilon-PVDF transfer membranes (Millipore, MA, USA) for immunoblotting. Nonspecific binding was blocked with 5% nonfat milk for 1 h at the room temperature. Bands were visualized by reacting with specific antibodies for DNMT1 (Santa Cruz biotechnology, CA, USA) and ERα (Millipore, MA, USA) overnight at 4°C. Sequentially, the anti-mouse (for DNMT1) or anti-rabbit (for ERα) secondary antibodies were conjugated to horseradish peroxidase and enhanced chemiluminescence was determined using Fuji medical X-ray film (Fujifilm, Tokyo, Japan).

### Cell proliferation and migration assay

To examine the effect of miR-152 on cell proliferation, HASMCs were transfected with the miR-152 precursor or inhibitor and then were incubated in microplates at 37°C with 5% CO_2_ for 48 h and 72 h. After that 0.5 mg/ml of dimethyl-thiazol- diphenyltetrazoliumbromide (MTT; Sigma-Aldrich, MO, USA) was added into each well. Spectrophotometric readings were performed by X340 spectrophotometer at 595 nm (Bio-TEK Instruments, Inc., VT, USA). Migration assays were performed following a standard protocol-wound repair assay. The mechanical injury of confluent HASMCs and lesion repair assay were performed as described elsewhere [42]. HASMCs were cultured in 6-well plates at 2×10^5^ cells/well as confluent monolayers. The monolayers were incubated and wounded in a line across the well with a 200-µl standard pipette tip. The wounded monolayers were washed twice with phosphate-buffered saline (PBS) and incubated with serum-containing medium supplemented with LPS. The rate of wound closure was measured and photographed over 24 h.

### Statistics

All values in the text and figures are expressed as mean ± SD. Statistical differences were evaluated by Student's t-test. A *p* value<0.05 was considered statistically significant.
